# Gelatin Meshes Enriched with Graphene Oxide and Magnetic Nanoparticles Support and Enhance the Proliferation and Neuronal Differentiation of Human Adipose-Derived Stem Cells

**DOI:** 10.3390/ijms24010555

**Published:** 2022-12-29

**Authors:** Aida Șelaru, Alexandra-Elena Mocanu-Dobranici, Elena Olăreț, Raluca-Elena Ginghină, Izabela-Cristina Stancu, Marieta Costache, Sorina Dinescu

**Affiliations:** 1 Department of Biochemistry and Molecular Biology, University of Bucharest, 050095 Bucharest, Romania; 2 Advanced Polymer Materials Group, Faculty of Chemical Engineering and Biotechnologies, University Politehnica of Bucharest, 011061 Bucharest, Romania; 3 Research and Innovation Center for CBRN Defense and Ecology, 041327 Bucharest, Romania; 4 Research Institute of the University of Bucharest (ICUB), 050663 Bucharest, Romania

**Keywords:** adipose-derived stem cells, fish gelatin, graphene oxide, magnetic nanoparticles, neuronal differentiation, tissue engineering

## Abstract

The field of tissue engineering is constantly evolving due to the fabrication of novel platforms that promise to stimulate tissue regeneration in the scenario of accidents. Here, we describe the fabrication of fibrous nanostructured substrates based on fish gelatin (FG) and enriched with graphene oxide (GO) and magnetic nanoparticles (MNPs) and demonstrate its biological properties in terms of cell viability and proliferation, cell adhesion, and differentiation. For this purpose, electrospun fibers were fabricated using aqueous precursors containing either only GO and only MNP nanospecies, or both of them within a fish gelatin solution. The obtained materials were investigated in terms of morphology, aqueous media affinity, tensile elasticity, and structural characteristics. The biological evaluation was assessed against adipose-derived stem cells by MTT, LDH, Live/Dead assay, cytoskeleton investigation, and neuronal *trans*-differentiation. The results indicate an overall good interaction and show that these materials offer a biofriendly environment. A higher concentration of both nanospecies types induced some toxic effects, thus 0.5% GO, MNPs, and GO/MNPs turned out to be the most suitable option for biological testing. Moreover, a successful neuronal differentiation has been shown on these materials, where cells presented a typical neuronal phenotype. This study demonstrates the potential of this scaffold to be further used in tissue engineering applications.

## 1. Introduction

Tissue engineering (TE) is a multidisciplinary field that seeks to reconstruct and restore the physiological function of damaged organs, emerging as an alternative to classical approaches such as tissue and organ transplants or autografts. These methods have been associated with various issues including immune rejection and immunosuppressive therapy, a lack of compatible donors, or donor site morbidity. Therefore, the current investigations have focused on developing alternative approaches that would overcome these challenges and could be used further in a clinical setting [1]. TE brings together knowledge from material and life sciences, aiming to develop tissue-like structures destined to support the regeneration process and acquire the normal physiological function. The major resources that are being used to accomplish this purpose include cells, scaffolds, and growth factors that regulate cellular processes such as proliferation or differentiation [2].

Considering the high variability of tissues and their different requirements and characteristics, it is of high interest to develop versatile scaffolds that would be suitable for a broad range of application. For this purpose, natural polymers such as fish gelatin (FG) have emerged as a promising resource. These can be obtained from various sources, have increased biocompatibility, low cytotoxicity, and antigenicity [2,3,4]. Gelatin is generated through the partial hydrolysis of native collagen, the major structural protein of extracellular matrix (ECM), and therefore inherits most of its biological properties. Aside from being highly biocompatible, gelatin is also biodegradable and presents bioactive motifs that mediate cell attachment and proliferation. As a result, it is a highly versatile polymer that can be used to develop scaffolds for multiple purposes such as wound healing, bone, nerve, and cardiac tissue regeneration [5,6,7,8]. FG comes as an alternative to the use of mammalian gelatin, which could be limited due to the risk of contracting certain diseases such as spongiform encephalopathy or even due to ethical and ethnocultural reasons [9,10]. Comparative analyses on both types of gelatins have revealed that FG could offer the same or even better results than mammalian gelatin in terms of biocompatibility and interaction with different cell types such as fibroblasts [11,12], preosteoblasts [13], or myoblasts [14]. These findings strengthen the idea that FG displays similar versatility and could be used as a substitute for mammalian gelatin in tissue engineering studies.

Current strategies to improve the interaction of the scaffolds with cells and the ability to support regeneration focus on developing and tailoring appropriate nanostructured architectures through nanoparticle enrichment. Numerous types of nanoparticles have been used for this purpose such as carbon-based, gold, ceramics, or even polymeric. It has been observed that nanopatterned scaffolds interact better with the cellular component, can regulate the cells’ behavior via physio-chemical cues, and provide a better spatiotemporal release of bioactive molecules. Moreover, nanoparticle loading is used to improve the mechanical properties of materials [15,16,17]. Graphene oxide (GO) is a carbon derivative used for scaffold functionalization and other biomedical applications due to its good physical and chemical characteristics such as electrical conductivity, high mechanical strength, and the ease of grafting different substitutes [18,19]. Studies on GO show promising results regarding the cytotoxic profile, cell attachment, and proliferation both in vivo and in vitro [20,21]. Moreover, scaffolds presenting GO enrichment were investigated for their ability to stimulate stem cell differentiation, especially toward osteogenic and neuronal lineage [21,22,23,24]. However, there are also reports on the positive effects of GO on hematopoietic [25], myogenic [26], and chondrogenic differentiation [27]. Additionally, magnetic stimulation and magnetic substrates have emerged as favorable cues in tissue engineering approaches [28,29], also focusing on bone and nervous tissue [30,31,32]. To achieve this, magnetic nanoparticle (MNP) loading is performed, displaying good biocompatibility and tailorable magnetic properties [33]. Studies on cellular morphology and cytoskeleton proteins show that magnetic cues induce the spatial reorganization of the cytoskeleton network, also having implications in the differentiation of stem cells [34]. Furthermore, MNPs could facilitate cell guidance and alignment for tissues possessing complex architectures, providing a detailed control over topographical and mechanical cues necessary to direct cell growth [35,36].

In terms of cellular resources, stem cells have become of high interest due to certain characteristics such as their relative ease to proliferate and populate the scaffolds and their ability to generate specialized cells of the body. Adipose-derived stem cells (ASCs) are adult mesenchymal stem cells with the potential to differentiate into numerous lineages such as adipogenic [37], chondrogenic, and osteogenic [30]. Moreover, ASCs are able to undergo differentiation toward cells with endodermal [38] or ectodermal origin [39]. Several studies have reported ASC differentiation toward neurogenic lineage using various biochemical and biophysical cues such as growth factors and inducers [40], magnetic [41], and electrical stimulation [39]. Aside from their great versatility, ASCs can be easily obtained through minimal invasive procedures from adipose tissue with relatively high yields, offering the perspective of personalized therapies, and thus being a valuable resource for TE applications [42].

Recent studies have described a good interaction between ASC- and GO-based substrates along with promising results during glial and neuronal differentiation, leading to the upregulation of specific markers such as glial-derived neurotrophic factor (GDNF), nerve growth factor (NGF), microtubule-associated protein 2 (MAP2), or NeuN [40,43,44]. Additionally, magnetic stimulation and magnetic scaffolds are used to promote the neuronal differentiation of adult stem cells including those of mesenchymal origin such as bone marrow derived stem cells (BMSCs) [45,46,47]. Therefore, in this study, we developed electrospun gelatin meshes containing different loadings of GO and MNP nanoparticles and combinations of both. Our aim was to investigate the in vitro effects of these nano-composites toward ASC behavior and determine whether the incorporation of both types of nanoparticles would lead to better outcomes in terms of proliferation, cellular morphology, and adhesion to the substrate. Given the contribution of GO and magnetic cues to neuronal differentiation, we also sought to assess whether our composites promote the ASC neuronal phenotype during *trans*-differentiation.

## 2. Results

### 2.1. Dynamic Light Scattering (DLS)

The hydrodynamic characteristics of each component and precursor are presented in Table 1. The high particle size distribution recorded for GO (Z-ave = 413.3 ± 50.44, PDI = 0.531 ± 0.028) can be attributed to its non-spherical shape, as previously reported [48,49]. The colloidal system comprised of both GO and MNPs suggests that between the two components, some interactions (probably hydrogen bonding) took place, leading to complexes with different hydrodynamic values (Z-ave = 460.5 ± 14.72, PDI = 0.581 ± 0.01) and higher stability, as expressed by ζ of −39.8 ± 0.889 mV. To understand the interactions between the precursor components, FG was introduced in the system at a concentration of 1 mg/mL. Under these conditions, the hydrodynamic parameters change due to the new interactions (electrostatic, hydrophobic, van der Waals, hydrogen bonding) that may occur between the components. The positive ζ of FG is attributed to the high number of amino groups present in its chemical structure.

### 2.2. Optical Microscopy (OM)

**Figure 1:**
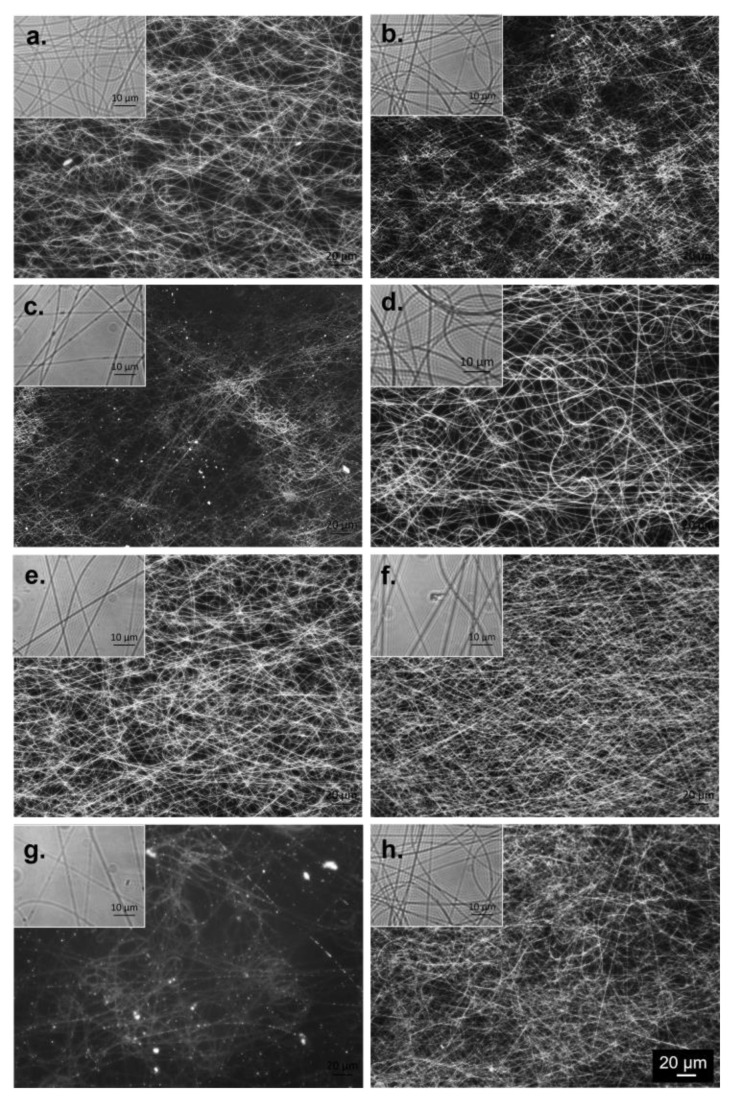
Representative optical microscopy images for the electrospun meshes: (**a**) FG, (**b**) FG_GO 0.5%, (**c**) FG_GO_1%, (**d**) FG_MNP 0.5%, (**e**) FG_MNP 1%, (**f**) FG_MNP 2%, (**g**) FG_GO_MNP 0.5%, (**h**) FG_GO_MNP 1%; general view 20×, scale bar 20 μm, the inset at the 100×-scale bar 10 μm.

OM indicated that all compositions were extruded into continuous and randomly oriented fibers (Figure 1). However, the organic–inorganic hybrid samples containing 1% *w*/*v* GO and the combination of both GO and MNPs generated fibers with visible agglomerates (white areas in Figure 1c,g) when compared to the other samples.

### 2.3. Scanning Electron Microscopy (SEM)

**Figure 2:**
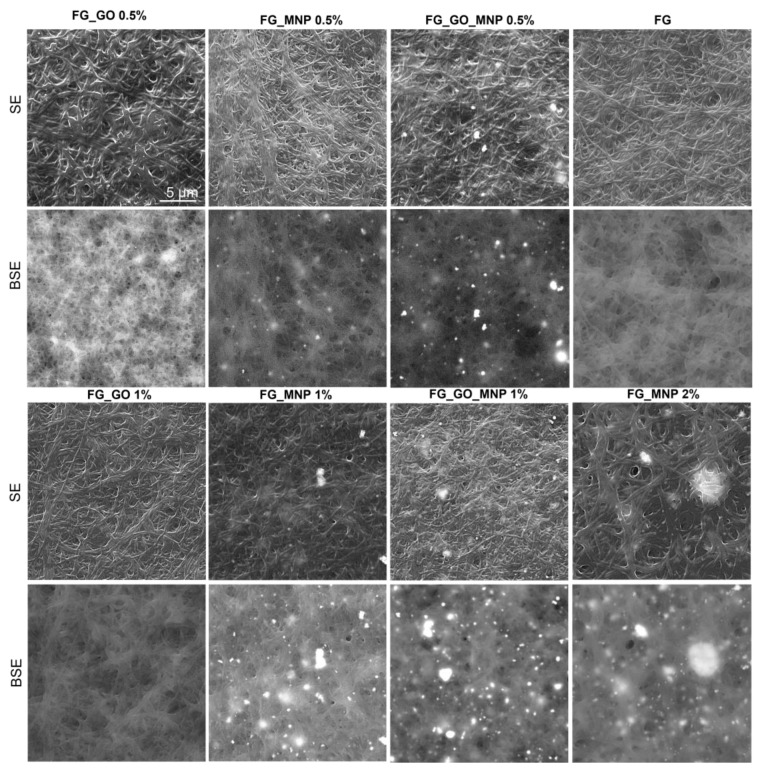
Representative SEM images for all samples after crosslinking (10 kX magnification). The scale bar of 5 μm is applicable for all images.

The SEM microanalysis revealed that the crosslinked scaffolds presented a typical fibrous microstructure regardless of the nanospecies used, with no significant differences between the compositions in terms of entanglement and fiber morphology. All individual fibers seemed to be continuous, smooth, and randomly oriented (Figure 2). FG_MNP_0.5% presented local parallel oriented bundles of fibers. The BSE micrographs highlighted the MNP distribution as bright areas due to the high atomic number of Fe, as previously reported in [50]. Increasing the MNP content led to agglomerates, with the highest tendency of clustering effect for the sample containing 2% MNP (Figure 2, BSE). Moreover, FG_MNP 0.5% presented the most homogeneous nanoparticle distribution when compared to the sample loaded with higher amounts of GO and MNP.

### 2.4. FTIR Spectrometry

Structural characterization through FTIR spectroscopy was performed on all the obtained meshes as well as on the raw protein (FG_raw). The FG_raw spectrum showed all of the specific peaks of proteins: a broad and intense peak at 3269 cm^−1^ assigned to amide A; amide B was noticed at 3085 cm^−1^; the doublet at 2995 cm^−1^ and 2935 cm^−1^ was assigned to saturated C–H stretching; amide I was mainly attributed to the stretching vibration of C=O and presented a strong peak at 1629 cm^−1^; amide II, related to the C–N stretch and N–H deformation vibrations, showed a peak at 1525 cm^−1^; and amide III at 1234 cm^−1^ was assigned to the C–N stretch, N–H bend, and CO in-plane bend.

The FTIR spectra of the organic–inorganic hybrids FG_GO, FG_MNPs, and FG_GO_MNPs suggest that the secondary structure of gelatin is not altered by the electrospinning process nor by the presence of nanospecies since all characteristic spectral bands were recorded at approximately the same wavenumbers when compared to the FG_raw spectrum. Only minor structural modifications were noticed. The spectrum of the FG fibers presented a slightly upshifted amide I to 1634 cm^−1^ due to hydrogen-bonding in the electrospinning precursor. The same influence was noticed through FG_GO, FG_MNPs, and FG_GO_MNP amide I and amide II.

### 2.5. Aqueous Media Affinity

**Figure 3:**
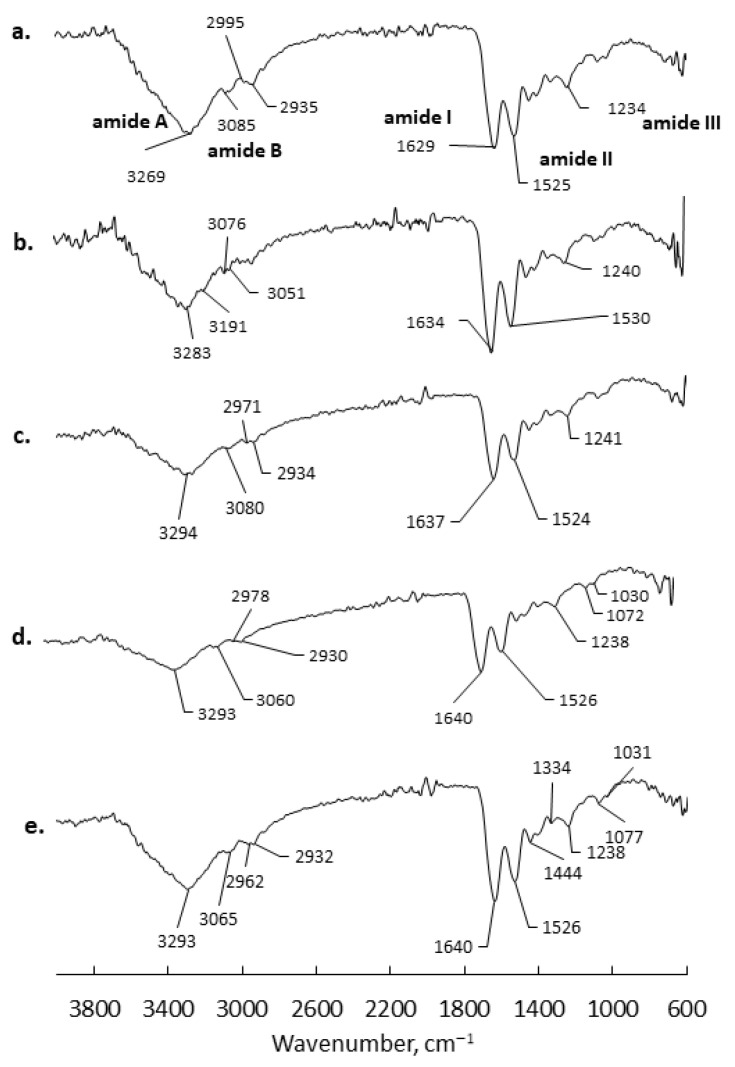
Representative ATR FTIR spectra recorded for the (**a**) raw fish gelatin, (**b**) FG fibrous mesh, (**c**) FG_GO mesh, (**d**) FG_ MNP mesh, (**e**) FG_GO_MNP.

The aqueous media affinity was evaluated through the swelling degree under the simulated physiological conditions (in PBS at 37 °C) and contact angle measurements (Figure 3). The maximum swelling degree was reached within 10 min for all compositions. No significant difference was observed when compared with the capacity of swelling in the PBS of FG mesh with FG_GO loaded meshes. However, the addition of 1% and 2% MNPs into the FG fibers led to higher maximum swelling degrees. When compared to the MNP loaded fibers, the highest MSD was reached when 1% MNP was used. Surface wettability confirmed, as expected, the hydrophilic nature of all materials. The addition of GO and MNPs increased the wettability of the FG mesh, with significant relevance for samples containing 2% MNP, and both combinations of GO and MNP. The lowest WCA value (69.49 ± 1.77) was obtained for FG loaded with 1% MNP, which also recorded the highest MSD (494.07 ± 5.59).

**Figure 4:**
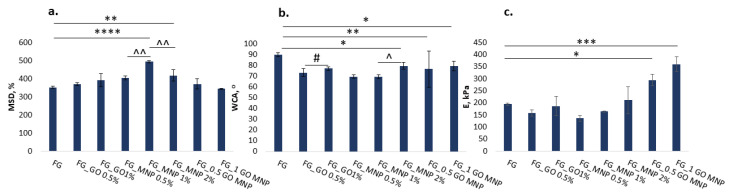
(**a**) Maximum swelling degree determined in PBS at 37 °C; (**b**) water contact angle; (**c**) tensile Young’s modulus at 5% deformation. Statistical significance: different symbols were used to show the statistical difference between different categories of samples- “*” was used to show comparison between the composites and the FG control; “#” was used for comparisons between samples with GO content; “^” was used for statistical comparisons between samples with MNP content; *, #, ^ *p* < 0.05, **, ^^ *p* < 0.01, *** *p* < 0.001, **** *p* < 0.0001.

The tensile elastic modulus (E) was computed at 5% deformation for each hydrated sample. The results show that E decreased with the addition of individual nanospecies up to 1%, resulting in more elastic samples. The double nano-structuring with GO and MNP led to an increase in E values, suggesting a synergistic reinforcement effect (Table 2/Figure 4c).

### 2.6. Biocompatibility Evaluation of Fibrous Gelatin Meshes

**Figure 5:**
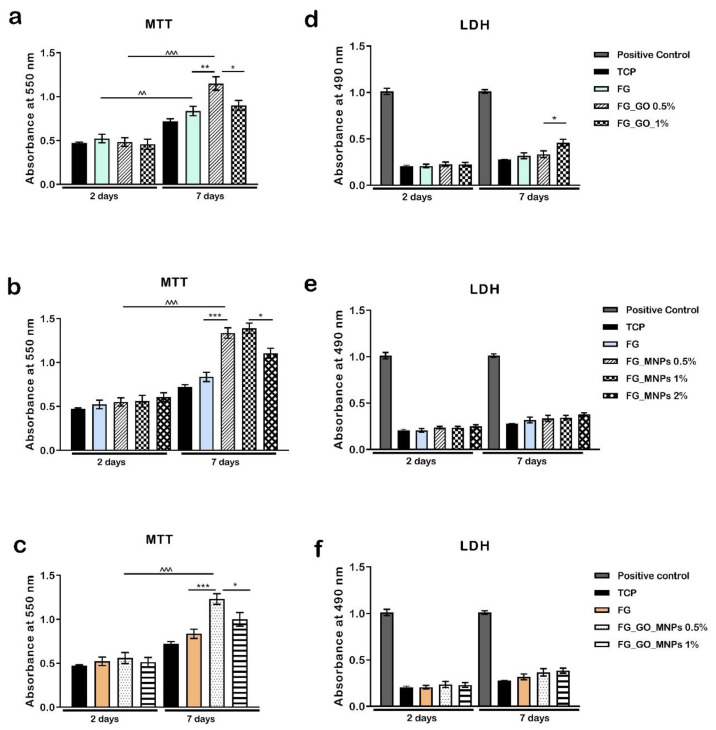
Biocompatibility evaluation of FG meshes in contact with mesenchymal stem cells. (**a**–**c**) Metabolic activity of hASCs as revealed by the MTT test during 7 days of in vitro cell culture. (**d**–**f**) Analysis of the materials’ toxicity by the LDH assay after one week of culture. Statistical significance: “*” symbol was used for statistical comparisons between different samples at the same time point, while “^” symbol was used to mark statistical differences between two different time points considering the same sample; * *p* < 0.05, ^^, ** *p* < 0.01, and ***, ^^^ *p* < 0.001.

Regarding the biocompatibility evaluation, the MTT profile at 2 days post-seeding revealed an overall good viability of hASCs in contact with these novel hybrid gelatin meshes (Figure 5). At this time point, no significant difference of cell viability was found between the tested composites, but a slightly higher rate was observed on hASC/FG_GO 0.5% compared to the hASC/FG_GO 1%. After 7 days of in vitro culture, the cells proliferated significantly on all scaffolds, with FG_GO 0.5% displaying the best results (*p* < 0.001).

Moreover, a significant difference in the cells’ metabolic activity was registered for the hASC/FG_GO 0.5% system, where the obtained values were higher in comparison to the ones found on FG (*p* < 0.01) and FG_GO 1% (*p* < 0.05). The decrease in the cells’ viability found on the FG_GO 1% composites suggests that increasing the GO concentration to 1% might have a negative impact toward cellular hASC culture. A similar behavior was observed for the behavior of hASCs in contact with gelatin meshes containing MNPs at 2 days of in vitro culture (b). However, for this set of nano-composites, small increases in the cell viability could be observed, proportional to the MNP concentration. After 7 days of culture, the hASC/FG_MNP 0.5% system displayed a significant proliferation from day 2 (*p* < 0.001) and a significantly increased viability compared to the hASC/FG system, while for the FG_MNPs 1% scaffold, the obtained values were slightly higher in comparison to the FG_MNPs 0.5% material, and a significantly lower viability rate was found on the FG_MNPs 2% composite (*p* < 0.05). We hypothesize that increasing the MNP concentrations have favorable effects on the hASC culture. However, a 2% concentration of MNPs might lead cells to proliferate too much, thus detaching from the substrate. Based on these observations, we eliminated the composites enriched with 2% MNPs as we did not notice any improvements in cellular behavior. Therefore, the last set of tested composites consisted of gelatin electrospun meshes with 0.5 and 1% GO and MNPs (c.). After preforming the MTT assay, the results indicate that systems with both GO and MNP loading generally display satisfactory viability and proliferation. In this case, it can be observed that the viability of the hASCs was significantly higher in FG_GO_MNPs 0.5% compared to the FG control and FG_GO_MNPs 1% (*p* < 0.01 and *p* < 0.05, respectively, at 7 days post-seeding). It could be that due to the addition of both nano-components, the concentration of 1% affected the cellular metabolic activity, thus registering a lower metabolic rate compared to the 0.5% GO and MNP scaffold.

For the evaluation of the cytotoxic effect of these materials upon the hASC culture, we addressed the LDH assay. All profiles revealed similar LDH levels between the tested materials, and comparable with levels in the case of hASCs cultured in a bidimensional system. For the FG_GO composites, it was observed that even though no significant differences were registered at the first time point, after 7 days of in vitro culture, the FG_GO 1% material displayed a significantly increased cytotoxicity (*p* < 0.05). Slightly higher values were registered for the FG_MNP and FG_GO_MNP meshes after one week and were proportional to the increase in nanoparticle concentration. However, they did not present significant differences in the LDH levels between the compositions at any time point. Therefore, a higher concentration of nano-composites does not induce significant cell damage or cell death, but at the same time, it also does not stimulate cell viability, thus not promoting a friendly environment for cell growth.

**Figure 6:**
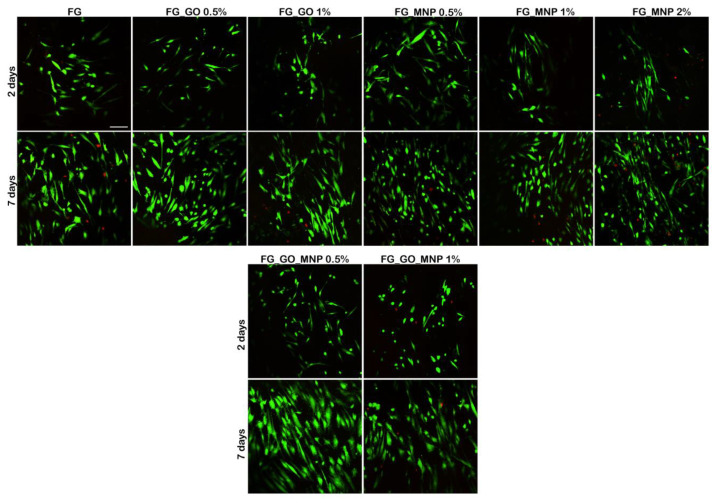
Biocompatibility evaluation of FG meshes in contact with human adipose-derived stem cells. Live/Dead staining on gelatin scaffolds shown through confocal microscopy: live cells are labeled in green fluorescence with calcein AM and the nuclei of dead cells is labeled in red with ethidium bromide. Scale bar = 50 µm.

Fluorescence images obtained after examination with confocal microscopy (Figure 6) revealed the overall high proportion of live, green labeled hASCs in contact with gelatin meshes, further supporting the idea that these materials support cell viability and proliferation.

It was noticed that at 2 days post-seeding, there were no significant differences in the shape of the cells and changes in phenotype. At this time point, they displayed a similar behavior, although it seems that in the presence of GO, they had a round shape, whereas in the presence of MNPs, they displayed a more elongated shape. Red nuclei indicate that dead cells were found at 2 days on the FG control, FG_GO 1%, and FG_MNPs 2%. Therefore, the addition of GO and MNPs improved the properties of the materials, but a higher concentration (1% GO, 2% MNPs) of these nanostructures caused the cell membrane to disrupt, thus exhibiting some toxic effects upon the hASC culture. The combination of GO and MNPs seems to have no extra influence upon the viability and cytotoxicity after 2 days of culture.

After 7 days of in vitro cell culture, Live/Dead staining evidenced an even distribution of the hASC population on these meshes. The proportion between green and red labeled cells remained similar to the one found at 2 days post-seeding. Furthermore, it was more obvious that the cells displayed a different shape according to the nano-component that had been added to the gelatin base. Therefore, on both FG_GO_0.5% and FG_GO_1%, the majority of the cells tended to present a round shape, in contrast to FG enriched with 0.5–2% MNPs, where the cells presented an elongated body. It was also noticed that a higher concentration of both GO and MNP produced several toxic effects, causing degradation of the membrane and leading to continuous cell death during a week of cell culture. It can only be assumed that on a longer term, the biological effect of these concentrations will not have beneficial outcomes upon cellular behavior.

A significant proliferation rate was found in all of the tested composites from 2 to 7 days of in vitro cell culture, especially for those enriched with GO and MNPs compared to the FG control, proving that the presence of these nanoparticles have a potent effect upon cell growth. After 7 days, a significant change was revealed on FG_GO_MNP 0.5% and FG_GO_MNP 1%.

### 2.7. Cytoskeleton Development of hASCs in Contact with Gelatin Fibrous Scaffold

**Figure 7:**
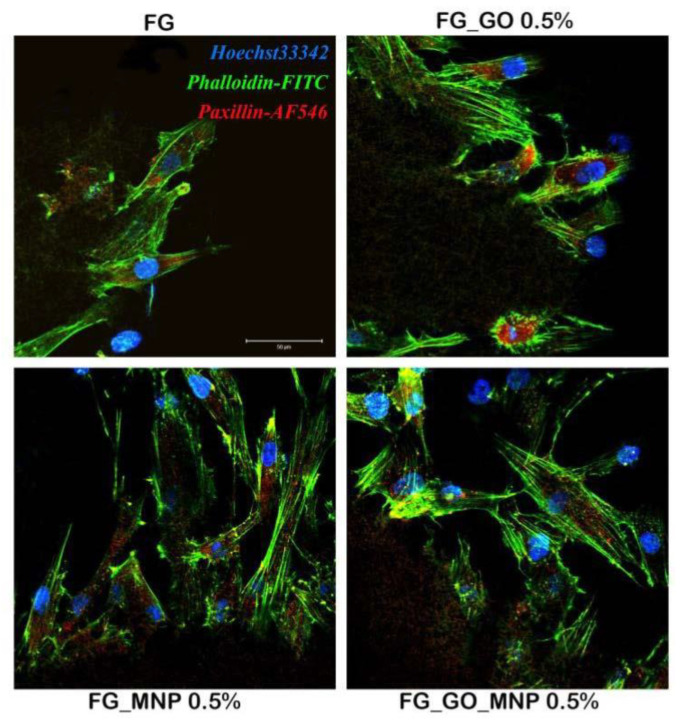
Development of the hASC cytoskeleton in contact with FG, FG_GO 0.5%, FG_MNP_0.5%, and FG_GO_MNP_0.5% after 48 h post-seeding. F-actin filaments are stained in green with phalloidin-FITC, paxilin in red with AF546, and cell nuclei are stained in blue with Hoechst 33342. Scale bar = 50 µm.

A good cell adhesion was observed after marking F-actin filaments with phalloidin-FITC (Figure 7). In all of the tested composites, a clear cytoskeleton with visible actin filaments could be noticed, together with the paxilin expression, which is normally found at the end of actin filaments, at the focal adhesion sites. A different manner of cellular adhesion was observed on these composites. Namely, on the surface enriched with GO, the hASCs displayed a round shape with paxilin expression found in a very different appearance compared to the one found on FG_MNP 0.5%, where the hASCs had elongated filaments with paxilin spread around the edges of the cells. This different behavior can be explained through the presence of the different nano-components present in the material. It could be that hASCs, in the presence of GO, accumulate around the graphene present in the material, therefore encouraging a round shape of the cytoskeleton. In contrast, in the presence of MNP, actin filaments in hASCs, together with paxillin, appear to adopt the elongated form. On the FG_GO_MNP 0.5% mesh, the staining revealed both round and elongated shapes of the hASC cytoskeleton. Overall, this proves that the material meets the criteria required to ensure optimal cell adhesion, a strong property that is essential when developing biomaterials for tissue engineering.

### 2.8. Neuronal Trans-Differentiation of hASCs in the Presence of Electrospun Meshes

**Figure 8:**
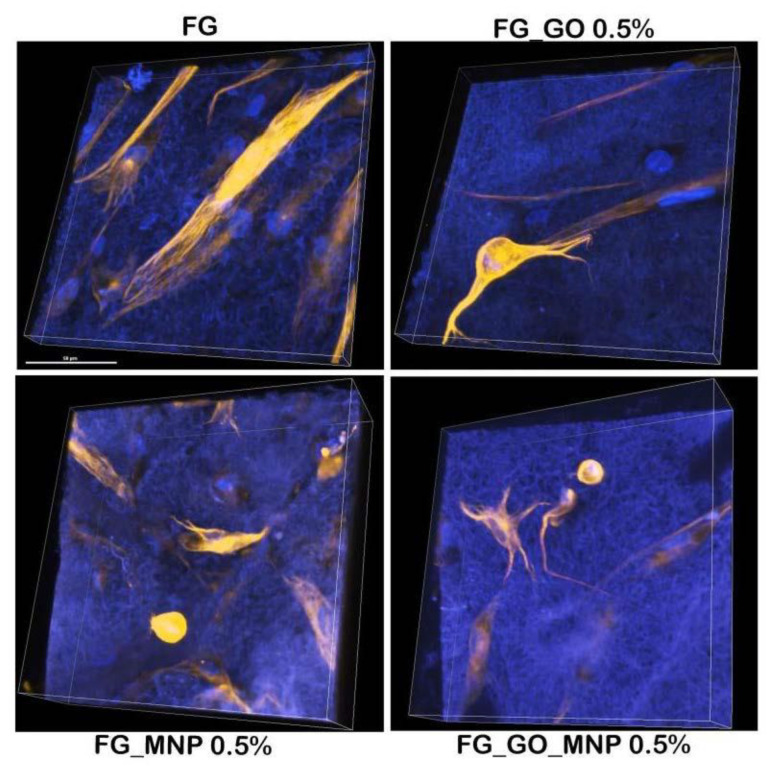
Immunofluorescence of the neuronal marker β-III-tubulin (marked with AF546 in orange, nuclei are marked with Hoechst33342 in blue) expressed in hASC/FG biosystems after 10 days of in vitro neuronal differentiation. Scale bar = 50 µm.

The biological studies were further expanded with a first evaluation of the potential of these biomaterials to support and stimulate the neuronal differentiation of hASCs. After the experiment was carried out, the immunofluorescence staining of neuronal maker β-III-tubulin revealed its expression after 10 days of induction in differentiated hASCs (Figure 8).

On the FG control, the expression of β-III-tubulin could be observed clearly, but no phenotypic changes were found on this material. In comparison, on the other meshes enriched with GO, MNPs, or both, there were morphological changes, namely, a round cell body with one, two, or more extensions, proving that the hASCs reached the neuron-like stage. The addition of these nano-components seemed to exhibit a positive effect on this kind of differentiation by stimulating these changes in the phenotype of hASCs. Thus, it has been demonstrated that these gelatin meshes exhibit unique biological properties and have multiple beneficial effects on hASC culture.

## 3. Discussion

Up to now, the tissue engineering field has emerged tremendously in terms of the fabrication of novel materials designed to substitute the damaged tissue. For this purpose, the use of a natural polymer and nanoparticles has always been attractive when designing new and original scaffolds. In this study, we propose some novel electrospun meshes enriched with graphene oxide and magnetic nanoparticles to serve as a substitute for multiple tissue engineering applications. All oof these components have shown incredible biological effects upon different cell types, recommending them for long-term use in tissue engineering applications. Moreover, a better understanding on the interaction between stem cells and these particular nano-components could shed more light in the field of tissue engineering and regenerative medicine.

Fish gelatin is a versatile polymer known to be biocompatible by its nature due to its high similarity to collagen, which is found in most tissues [8]. Gelatin has been tested as a candidate for several tissue engineering applications, among them bone [51], adipose [52], muscular [53], and nervous tissue engineering [54]. In this study, we obtained similar results as the hASCs also displayed a good interaction with the plane control material, FG. Over the last decade, GO has gained in popularity not only in the field of regenerative medicine, but also in drug delivery systems. Due to its unique physiochemical properties, it can interact with cells in a bio-friendly manner, if the concentrations are kept under control. Server studies [55,56,57] have demonstrated that a higher concentration of GO (up to 3%) has inhibiting effects on cell growth and may exhibit toxic effects long-term. MNPs, incorporated in biopolymers as a nano-component, have demonstrated a beneficial environment for cell growth and proliferation in many cases, but especially for stem cells [33]. It seems that these particles have an interesting way in which they can control the spatiality and growth of stem cells [34]. It has been demonstrated that the magnetic field generated by these particles has helped in the differentiation of stem cells toward osteogenic [30], chondrogenic [58], cardiac [59], and neuronal lineages [60].

Starting from our previous studies [61,62,63], we fabricated random oriented nano-composite fibers based on FG as a polymeric matrix loaded with GO–COOH, MNPs, and both, and further characterized them in terms of aqueous media affinity, tensile elasticity, and biocompatibility. The overall stability and performance of the fabricated scaffolds are affected by the interactions that occur between the components of each composition. Hence, qualitative information about these interactions was obtained through DLS measurements. The best stability and dispersibility was obtained for MNPs, while GO showed good stability but a high polydispersity, which was attributed to its non-spherical shape [64]. When the two were put together, the mean hydrodynamic size and polydispersity increased (z-ave = 460.5 ± 14.72, PdI = 0.581 ± 0.01) as well as the zeta potential (ζ = −39.8 ± 0.889). This suggests that some of the monodispersed MNPs adsorbed onto the GO surface increased the stability of the bicomponent system. Changes that appeared in all hydrodynamic parameters when the protein was added to the system suggest that a combination of interactions (electrostatic interactions, hydrogen bonding) take place between the components, changing the conformation of the individual component [65]. The obtained fibrous substrates showed continuous, smooth fibers with a better MNP when lower concentrations were used (FG_MNP 0.5%).

The affinity to aqueous media is an important property that influences cell adhesion and material integration into the host tissue, and it was gravimetrically evaluated through the maximum swelling degree in PBS at 37 °C and surface wettability through contact angle measurements. As expected, due to the hydrophilic nature of the precursors, the composite scaffolds maintained this property. As can be observed in Figure 2, the swelling ability in PBS was slightly increased by the addition of nanostructures, probably due to a different arrangement of macromolecule chains. Moreover, the presence of nanostructures might consume some functionalities that would otherwise have been available and consumed in the crosslinking process, leading to a more robust network with less swelling capacity, as observed for the FG sample. Although the surface wettability (against distilled water) was slightly improved by the presence of nanoparticles, it could be observed that with an increase in the nanoparticle concentration, the effect on wettability decreased. This behavior can be influenced by many other factors derived from the electrospinning process (such as fiber diameters or fiber homogeneity, which may vary from one composition to another) or from the crosslinking processes.

Mechanical properties are of great importance and should be suitable for the final application of the materials. Aside from the chemical cues or specific morphology, it has been proven that mechanical properties have a significant role in cellular activity and behavior [66]. The tensile modulus of the fabricated meshes was found to be between 135 and 360 kPa including them in the soft tissue range [67]. Our results showed that the addition of 0.5% or 1% GO or MNPs led to more elastic fibrous substrates when compared to the FG mesh. Samples containing 1% nanoparticles were slightly stiffer than their 0.5% counterparts, while samples containing 2% *w*/*v* MNPs, 0.5% *w*/*v* MNPs and GO, and 1% *w*/*v* MNPs and GO increased the E with approximately 8.5%, 51.5%, and 86.6%, respectively.

The performed biocompatibility assays (MTT, LDH, and Live/Dead) demonstrated that both the GO and MNP incorporated materials had a positive influence on the cell viability rate. The level of cytotoxicity found on the composites was not significant, indicating that relatively low concentrations of GO and MNPs do not induce a toxic effect on hASCs. On the other hand, for the FG_GO 1% material, a significant increase in the LDH levels was registered at 7 days compared to FG_GO 0.5%, suggesting that a concentration of up to 1% negatively affects the hASC culture. Indeed, there are concerns regarding GO toxicity, and it was proven that the cytotoxic effects could be decreased through hybridization with biopolymers [68]. Moreover, the elevated cytotoxicity also correlated with changes in the cells’ metabolic activity at the same time point, since increasing the GO concentration to 1% led to a significant diminishing in the viability of hASCs. A similar profile was observed for the MNP enriched materials where the hASC/FG_MNP 2% system displayed a decreased viability, but with no significant differences in terms of cytotoxicity compared to other MNP loadings at 7 days. In parallel, the MTT assay revealed that the addition of MNPs and GO (0.5%) on the biomaterials significantly increased the viability of the mesenchymal stem cells during one week of in vitro cell culture compared to the control. Simultaneously, the labeling of live and dead cells confirmed the high proliferation rate of hASCs, and there were fewer red labeled nuclei in contact with FG_GO 0.5% and FG_MNPs 0.5% up to 7 days in culture.

Additionally, fluorescent microscopy revealed the formation of cell groups at 7 days post-seeding, demonstrating that the material offers optimal conditions for hASCs to grow and proliferate. In the case of hASC/FG_MNP systems, Live/Dead images revealed an elongated morphology of the cells, which were stretched along the substrate, which can be correlated with the fact that the dispersability of the MNPs was higher than the one in the case of GO. Such behavior is consistent with the literature data reporting the influence of magnetic cues causing cytoskeleton strain and focal adhesion rearrangements [34,57]. These changes were also reported during differentiation of mesenchymal stem cells toward chondrogenic and tenogenic lineages [34,57]. In the study conducted by Tomás et al. (2018), hASCs grown on a magnetic substrate presented the reorganization of actin microfilaments with an anisotropic distribution. Furthermore, systems containing both GO and MNP loading displayed a different behavior depending on the nanoparticle concentration, with the 0.5% enriched materials providing the most suitable environment for hASC culture. As observed above, the materials’ structure and properties changed when GO and MNPs were combined, thus we expected the cells to change their behavior. Therefore, the addition of GO and MNPs could be used as a modulating tool of cellular behavior in terms of viability and proliferation. We expected that the addition of MNPs, aside from GO content, would help diminish its cytotoxicity since MNPs possess a higher biocompatibility and lower toxic effects. However, at the 1% concentration, these composites did not determine better outcomes, as can be seen from biocompatibility assay results.

These observations were further validated through the cytoskeleton investigations, which revealed an interesting interaction between the cells and the surface of the material. Different cellular behavior was observed between the two types of nano-components used in these studies. In the presence of MNPs, overall, the hASCs presented an elongated shape, whereas in the presence of GO, they had the tendency to adopt a round shape. The biofriendly environment that these nano-components offer is not questionable, but depending on the formulation and the overall fabrication of the material, the cells may respond in a different manner to their presence. This could also be observed in this case, where two different responses of the same cell types were highlighted. Thus, depending on the desired application, cells could be directed correspondingly by manipulating the type of nano-component.

For the *trans*-differentiation studies, our results indicated a successful achievement of neuron-*like* cells derived from hASCs. The neuronal differentiation of stem cells in the presence of graphene oxide has been widely studied in the last years [69]. Phenotypic changes occurred during the *trans*-differentiation of hASCs in the presence of GO compared to the control of the experiment, proving that the induction medium alone is not enough. Thus it can be stated that GO possesses a neuroinductive effect. This effect has been strengthened by Wang et al. [70] via functionalization with fluorine groups. These changes have also been noted to occur in the presence of MNPs. Other studies [71,72] have conducted similar studies and demonstrated the neuronal effect of these particles. These results open lots of possibilities to further explore the relationship between these nano-components and hASCs for more sophisticated TE applications such as nervous tissue engineering. This first dataset proves the potential of both the hASC and FG_GO_MNP platforms to bring new advantages to this field, and we aim to conduct future studies that will investigate the molecular cues that take place in hASCs and these materials during neuronal *trans*-differentiation.

The combination of these components is the novelty of this study and to our knowledge, we present the first results that indicate the combination of GO and MNPs on the same platform exhibit an enhanced effect upon viability, proliferation, and cell differentiation.

## 4. Materials and Methods

Gelatin (FG) from cold water fish skin, graphene oxide (GO) (Graphenea), magnetite nanopowder (MNP), absolute ethanol (Chemical company, Iași, Romania), glutaraldehyde as a 25% *w*/*v* aqueous solution, and phosphate buffered saline (PBS) were used. All reagents were purchased from Sigma-Aldrich if not otherwise mentioned. For all compositions, double distilled water was used (obtained using a GFL 2021, Burgwedel, Germany), sterile filtered through a 0.22 μm syringe PES filter.

### 4.1. Fabrication of Fibrous Meshes through Electrospinning

Electrospinning precursors were prepared by adapting the protocols described in [60]. Suspensions of MNP and GO were prepared in double distilled water to the concentrations indicated in Table 3. In brief, each nanoparticle suspension (see Table 3) was sonicated for 2 h using a laboratory ultrasonic processor—UP100H (Hielscher—Ultrasound Technology, Germany) to assure a homogenous distribution of the nanospecies. Then, FG was gradually added up to a final concentration of 50% *w*/*v* and left to dissolve under vigorous magnetic stirring at 40 °C for 4 h.

The fibrous meshes were fabricated using climate-controlled electrospinning (EC-CLI, IME Medical Electrospinning, Waalre, The Netherlands). Each electrospinning precursor was loaded in a 5 mL syringe connected to a pump and ejected with a precisely controlled flow rate until a final volume of 800 μL. The applied voltage was varied between 19 kV and 25 kV depending on the precursor’s formulation. The temperature and relative humidity were set to 25 °C and 40%, respectively. Fibers were collected on a 90 mm diameter drum, rotating at 100 rpm. The detached fibrous scaffolds were crosslinked in an ethanolic glutaraldehyde solution with a 1% *w*/*v* concentration for 4 days, washed with ethanol for 2 days, and with distilled water for another 2 days.

### 4.2. Dynamic Light Scattering (DLS)

The mean hydrodynamic diameter (Z-ave), polydispersity index (PdI), and zeta potential (ζ) measurements were achieved by a dynamic light scattering (Zetasizer Nano ZS, Malvern Instrument, Worcestershire, UK) device equipped with a He/Ne laser. Size measurements were performed at a scattering angle of 173° while the zeta potentials were converted from the electrophoretic mobility operating at an angle of 13° using the Helmholtz–Smoluchowski equation. The analyzed suspensions (GO, MNPs) were prepared at a concentration of 0.125 mg/mL. The bi and tri- component systems were prepared by mixing 1:1 *v*/*v* of each suspension with 1 mg/mL FG solution.

### 4.3. Optical Microscopy

The fibrous structures were analyzed through optical microscopy (OM) using a Zeiss Axiovert microscope with an Axiocam 503 monochrome.

### 4.4. Scanning Electron Microscopy (SEM)

Morpho-structural characteristics of the crosslinked meshes were evaluated through SEM images using a Tescan Vega II LMU SEM at 30 keV, and at high vacuum of 3.4 × 10^−2^ Pa using a SE detector and VegaTC software. The device was equipped for EDS microanalysis with a Bruker Quantax XFlash 6/10 energy-dispersive X-ray spectrometer. Micrographs were recorded in the secondary electron (SE) and backscattered electron (BSE) modes. Prior to image acquisition, the samples were coated with Au/Pd under Ar plasma using a Quorum minisputter SC7620, under an argon chamber pressure at 2 × 10^−1^ mbar for 3 min at a 1.5 mA process current.

### 4.5. FTIR Spectrometry

Structural characterization of the electrospun fibrous substrates was evaluated through attenuated total reflectance Fourier transform infrared (ATR-FTIR) using a JASCO 4200 (Deutschland GmbH, Pfungstadt, Germany) spectrometer with a Specac Golden Gate ATR. For all spectra, 200 scans were recorded at a resolution of 4 cm^−1^, in the wavenumber region of 4000–600 cm^−1^. MNP, GO, and FG were used as control samples.

### 4.6. Contact Angle and Swelling Degree

The aqueous media affinity was monitored through the water contact angle (WCA, °) measurements and maximum swelling degree (MSD, %) in phosphate buffer saline (PBS) at pH 7.4. For wettability measurements, Drop Shape Analyzer 100 (DSA 100, KRÜSS GmbH, Hamburg, Germany) equipment was used to dose ~2 μL of double distilled water and placed on a mesh surface. The drop shape was captured using a high-resolution camera and WCA was computed according to the Young–Laplace equation. At least three measurements were performed on different locations for each sample and the results were expressed as the mean ± standard deviation. MSD was gravimetrically determined as described in [62], using Equation (1), where *m_f_* represents the weight of the sample at the equilibrium swollen state and *m*_0_ is the initial weight of the dry sample.

(1) $$MSD,\;\%=\frac{m_{f}-m_{0}}{m_{0}}\;\times \;100$$

### 4.7. Uniaxial Tensile Tests

Mechanical properties were investigated through uniaxial tensile tests using a Brookfield CT3 texture analyzer with a 45 N load cell. Fully hydrated specimens were cut into rectangular shapes of 30 mm × 10 mm. Specimens with an active area of 20 mm × 10 mm were used (a 5 mm length of each sample’s end was used for gripping). Samples were tested with 0.5 mm/s until 90% deformation was reached. At least three specimens of each sample were tested and used to compute the tensile elastic modulus (*E*, kPa) at 5% deformation according to Equation (2), where *σ* is the applied stress and *ε* is the strain.

(2) $$E=\frac{\sigma }{\varepsilon }$$

### 4.8. Biocompatibility Evaluation of Fibrous Gelatin Meshes

In order to monitor the biological effects of gelatin meshes enriched with GO, MNPs, and both, they were put in contact with human adipose-derived stem cells (hASCs). The samples were cut to the dimensions of 1.6 cm^2^ in diameter and placed in 24-well plates. Next, they were exposed to UV light, washed three times with PBS and placed in the last PBS bath with 3% antibiotic overnight. Next, the hASCs (StemPro Human Adipose-Derived Stem Cells, ThermoFisher Scientific) were expanded in culture following the manufacturer’s instructions, in MesenPro RS Basal Medium (ThermoFisher Scientific), supplemented with MesenPro Growth Supplement and L-glutamine. Cells in passage 4 were seeded on gelatin meshes at a density of 2 × 10^4^ cells/cm^2^, thus resulting in 2D cell–scaffold systems, further named hASCs/FG_GO, hASCs/FG_MNPs, and hASCs/FG_GO_MNPs. These were maintained in standard culture conditions (37 °C, 5% CO_2_, and humidity) for 7 days, during which biocompatibility assays (MTT, LDH, Live/Dead) were carried out at 2- and 7-days post-seeding, and the cell adhesion assessment was performed at 48 h after seeding.

The metabolic activity of living cells in contact with FG-based meshes was assessed using methylthiazolyldiphenyl tetrazolium bromide (MTT, Sigma-Aldrich Co., Steinheim, Germany). The solution was prepared at the recommended concentration of 1 mg/mL in PBS. After 4 h of incubation with the MTT solution, the resulting formazan crystals were dissolved using isopropanol to a final violet solution. The hASCs at a density of 2 × 10^4^ cells/cm^2^ on 24-well plates served as a positive control of cell viability. The obtained solution was measured at 550 nm using a FlexStation3 spectrophotometer (Molecular Devices, San Jose, CA, USA).

The cytotoxic effect of the scaffold was assessed using the “In vitro toxicology assay kit lactate dehydrogenase based” TOX7 Kit (Sigma Aldrich Co., Steinheim, Germany). The positive control for this assay was represented by the hASCs seeded on the culture plates, which were treated with 2% Triton-X100 (Sigma/Merck, Steinheim, Germany) in order to induce 100% of cell cytotoxicity. The test was performed following the manufacturer’s instructions and the final product was measured at 490 nm using a FlexStation3 spectrophotometer (Molecular Devices, San Jose, CA, USA).

To qualitatively observe the living and dead cells in contact with the scaffolds, the staining was performed using the Live/Dead Kit (Invitrogen, Life Technologies, Foster City, CA, USA). The staining solution was prepared following the manufacturer’s instructions and 300 µL of staining solution was added on each well and incubated for 20 min in the dark at room temperature. The examination was performed using a laser-scanning confocal microscope (Nikon A1/A1R Confocal Laser Microscope System) and images were analyzed using the corresponding software.

After the biocompatibility evaluation, only the composites with the best results were selected for further biological investigations.

### 4.9. Cytoskeleton Development of hASCs in Contact with Gelatin Fibrous Scaffold

In order to investigate cell adhesion on these gelatin meshes, F-actin filaments together with paxillin were evidenced 48 h post-seeding. For fixation of the cells, a 4% paraformaldehyde solution (Sigma Aldrich Co., Steinheim, Germany) was used for 1 h. Then, the cell membrane was permeabilized with 0.1% Triton X-100 (Sigma Aldrich Co., Steinheim, Germany) and 1.2% bovine serum albumin (BSA) solution for 45 min. The samples were incubated with paxillin antibody (Sigma/Merck, Steinheim, Germany) for 12 h at 4 °C, after which they were incubated with anti-mouse Alexa Flour 546 secondary antibody (Invitrogen) for 2 h at RT and in the dark. Next, cell–scaffold systems were incubated for 1 h with phalloidin-FITC (Sigma Aldrich Co., Steinheim, Germany) and for 5 min with Hoechst 33342 (ThermoFisher Scientific, Foster City, CA, USA) in order to stain the cell nuclei. A laser-scanning confocal microscope (Nikon A1/A1R Confocal Laser Microscope System) was used for visualization and the images were analyzed using corresponding software.

### 4.10. Neuronal Trans-Differentiation of hASCs in the Presence of Electrospun Meshes

To further test the biological effects of these meshes, we induced the neuronal differentiation of hASCs in contact with FG meshes during 10 days of culture. For this purpose, hASCs were seeded on FG samples at a density of 2 × 10^4^ cells/cm^2^ and after 24 h, they were treated with the corresponding differentiation media (PromoCell, Heidelberg, Germany) for 7/10 days. The medium was changed every 2 days. The expression levels of the neuronal maker, β-III tubulin, was evaluated by immunofluorescence and confocal microscopy. After fixation and permeabilization, the samples were incubated with β-III tubulin antibody (Cell Signaling Technology, Danvers, MA, USA) overnight at 4 °C and then incubated with anti-rabbit Alexa Flour 546 secondary antibody (Invitrogen) for 2 h at RT and in the dark. Finally, the samples were incubated with Hoechst 33342 (ThermoFisher Scientific, Foster City, CA, USA) for 5 min in order to show the cell nuclei. A laser-scanning confocal microscope (Nikon A1/A1R Confocal Laser Microscope System) was used for visualization and the images were analyzed using the corresponding software.

### 4.11. Statistical Analysis

All experiments were performed in triplicate (n = 3) and the results were expressed as a mean ± standard deviation (SD) using GraphPad Prism Software 6.0 (GraphPad Software Inc., San Diego, CA, USA). Statistical relevance was assessed using the same software, one-way ANOVA method, and Bonferroni post-test and significant statistical differences were considered for *p* < 0.05.

## 5. Conclusions

The biological interaction between hASCs and FG_GO/MNP meshes has been an effective one. The incorporation of GO and MNPs in low concentrations did not affect the cell viability and proliferation. The aim of this study was to explore the potential modulatory effect of GO and MNPs incorporated in fish gelatin meshes on cellular activity. The addition of both GO and MNPs significantly increased the tensile Young’s modulus, leading to more rigid networks when compared to the FG mesh. These changes in the structure of the material had a direct effect upon cell morphology and behavior with the best results for the concentration of 0.5% GO, MNPs, and GO/MNPs. The hASCs responded differently in the presence of GO and MNPs, as evidenced through the evaluation of the cytoskeleton, thus proving the versatility of these materials and their potential use for any desired TE application. Here, we explored the potential for nervous tissue engineering by addressing the neuronal *trans*-differentiation of hASCs in the presence of these scaffolds. The expression of the neuronal maker β-III tubulin was found in contact with all of the tested composites, but morphological changes in the differentiated hASCs were only found on the platforms enriched with GO, MNPs, and GO/MNPs. Thus, it can be considered that they also possess neuronal modulation properties. Further investigations need to be carried out in order to confirm this hypothesis.

## Figures and Tables

**Table 1:** The hydrodynamic parameters of each component and precursor system.

Sample	Z-Ave, nm	PDI	ζ (mV)
MNPs	139.9 ± 7.559	0.273 ± 0.006	−36.4 ± 0.666
GO	413.3 ± 50.44	0.531 ± 0.028	−30.7 ± 0.723
GO–MNPs	460.5 ± 14.72	0.581 ± 0.01	−39.8 ± 0.889
FG	381.3 ± 15.39	0.499 ± 0.018	+8.23 ± 0.071
MNPs_FG	296.6 ± 2.15	0.511 ± 0.008	+0.0168 ± 0.0083
GO_FG	346.6 ± 6.59	0.435 ± 0.023	+7.04 ± 0.0757
GO_MNPs_FG	399.2 ± 0.5859	0.497 ± 0.021	+1.27 ± 0.0115

**Table 2:** Summarized results of the contact angle measurements, maximum swelling degree, and tensile Young’s modulus.

Sample	WCA, °	MSD, %	E, kPa
FG	89.68 ± 1.82	351.70 ± 6.56	194.18 ± 4.48
FG_GO 0.5	73.22 ± 3.75	370.00 ± 8.72	156.86 ± 13.06
FG_GO 1	77.13 ± 1.62	392.25 ± 35.60	186.67 ± 39.07
FG_MNPs 0.5	69.62 ± 1.38	403.81 ± 10.03	135.58 ± 12.06
FG_MNPs 1	69.49 ± 1.77	494.07 ± 5.59	163.78 ± 1.95
FG_MNPs 2	79.48 ± 3.17	418.06 ± 30.36	210.84 ± 55.86
FG_GO_MNP 05	76.49 ± 16.62	371.43 ± 26.96	294.32 ± 23.04
FG_GO_MNP 1	79.34 ± 4.30	344.44 ± 3.70	360.41 ± 31.21

**Table 3:** Detailed formulations of the precursor compositions (protein concentration was 50% *w*/*v* in all compositions).

Sample Name	MNP, *w*/*v*	GO, *w*/*v*
FG	0	0
FG_MNP 05	0.5	0
FG_MNP 1	1	0
FG_MNP 2	2	0
FG_GO 05	0	0.5
FG_GO 1	0	1
FG_MNP 05_GO 05	0.5	0.5
FG_MNP 1_GO 1	1	1

## Data Availability

Not applicable.
